# Invited Response on: Adipose Tissue in Multiple Symmetric Lipomatosis Shows Features of Brown/Beige Fat

**DOI:** 10.1007/s00266-021-02670-0

**Published:** 2021-11-15

**Authors:** Daniel Schiltz, Stephan Schreml

**Affiliations:** 1 Department of Plastic, Hand- and Reconstructive Surgery, University Hospital Regensburg, Regensburg, Germany; 2 Department of Dermatology, University Hospital Regensburg, Franz-Josef-Strauss-Allee 11, 93053 Regensburg, Germany

*Level of Evidence V* This journal requires that authors assign a level of evidence to each article. For a full description of these Evidence-Based Medicine ratings, please refer to the Table of Contents or the online Instructions to Authors http://www.springer.com/00266.

Dear Xinhang Dong et al.,

Thank you very much for your thoughts on this study [1]. The hematoxylin-eosin staining of affected and unaffected fatty tissue in patients suffering from MSL showed increased inflammatory infiltration as compared to control fatty tissue of healthy patients. However, we found that overall there was no difference between affected and unaffected tissue in MSL patients (although macrophages were not counted). These results are consistent with those obtained by CD200 and UCP1 immunohistochemistry. Evaluation of table 1 was subjective, as we do not have an automated (objective) quantification. This is a limitation of the study. We thank the authors for the elaboration on the different types of macrophages (especially M2-type) that have different phenotypic characteristics and biological functions. Further evaluation regarding M2 macrophages would be a current and interesting topic for further research in our patient cohort.
